# Stable and unstable miscible displacements in layered porous media

**DOI:** 10.1017/jfm.2019.190

**Published:** 2019-04-25

**Authors:** Japinder S. Nijjer, Duncan R. Hewitt, Jerome A. Neufeld

**Affiliations:** 1 Department of Applied Mathematics and Theoretical Physics, University of Cambridge, Wilberforce Road, Cambridge CB3 0WA, UK; 2 Bullard Laboratories, Department of Earth Sciences, University of Cambridge, Madingley Road, Cambridge CB3 0EZ, UK; 3 BP Institute, University of Cambridge, Madingley Road, Cambridge CB3 0EZ, UK

**Keywords:** fingering instability, mixing and dispersion, porous media

## Abstract

The effect of permeability heterogeneities and viscosity variations on miscible displacement processes in porous media is examined using high-resolution numerical simulations and reduced theoretical modelling. The planar injection of one fluid into a fluid-saturated, two-dimensional porous medium with a permeability that varies perpendicular to the flow direction is studied. Three cases are considered, in which the injected fluid is equally viscous, more viscous or less viscous than the ambient fluid. In general it is found that the flow in each case evolves through three regimes. At early times, the flow exhibits the concentration evolves diffusively, independent of both the permeability structure and the viscosity ratio. At intermediate times, the flow exhibits different dynamics including channelling and fingering, depending on whether the injected fluid is more or less viscous than the ambient fluid, and depending on the relative magnitude of the viscosity and permeability variations. Finally, at late times, the flow becomes independent of the viscosity ratio and dominated by shear-enhanced (Taylor) dispersion. For each of the regimes identified above, we develop reduced-order models for the evolution of the transversely averaged concentration and compare them to the full numerical simulations.

## 1 Introduction

Miscible displacement processes, in which a viscous fluid is injected into a fluid-saturated porous medium, are relevant in a number of problems including enhanced oil recovery (Lake 1989), carbon capture and storage (Huppert & Neufeld 2014) and subsurface contaminant transport (Abriola 1987). In many of these cases, the goal is to optimize and control the displacement front and the amount of mixing that occurs between the two fluids. The evolution of the displacement front is controlled by a number of factors; in this paper we will examine the combined effects of both permeability heterogeneities and viscosity variations.

If the porous medium is homogeneous and the injected and ambient fluids have different viscosities then, depending on the viscosity ratio, the displacement front can be stable or unstable. If the injected fluid is more viscous, the interface is stable and the fluid front moves uniformly, whereas if the injected fluid is less viscous the displacement front can be unstable and lead to complex fingering patterns. Miscible viscous fingering has been extensively studied using a number of different approaches (for example Tan & Homsy 1986, 1987, 1988; Zimmerman & Homsy 1991, 1992; Jha, Cueto-Felgueroso & Juanes 2011*a*,*b*; Chui, De Anna & Juanes 2015; Pramanik & Mishra 2015*b*). Recently, Nijjer, Hewitt & Neufeld (2018) described the full lifecycle of miscible viscous fingering: they identified the different regimes through which the flow evolves and in each regime modelled the evolution of the flow. We take a similar approach in this paper and look at the role of permeability heterogeneities on the temporal evolution of the displacement front in the case of neutral, stable and unstable displacements.

Most physically relevant porous media are not homogeneous but vary on a wide range of length scales from the pore scale to the reservoir scale in both an ordered and disordered manner (Weber 1986). A number of studies have considered the combined effects of both randomly varying permeability fields and viscosity variations on miscible displacements using theoretical (Welty & Gelhar 1991), numerical (Tan & Homsy 1992; Waggoner, Castillo & Lake 1992; Chen & Meiburg 1998; Camhi, Meiburg & Ruith 2000; Talon *et al*. 2004; Tchelepi *et al*. 2004; Nicolaides *et al*. 2015) and experimental (Jiao & Hotzl 2004; Tchelepi *et al*. 2004) approaches. These studies have demonstrated that the flow exhibits a range of dynamical behaviour, including dispersing, channelling and fingering, and highlight the complexity of the flow patterns that arise.

In this work, we focus on large-scale permeability variations that are perpendicular to the flow direction. This structure is widespread in nature, being characteristic of geological formations consisting of different sedimentary sequences. Previous work looking at the combined effect of permeability variations and an unstable flow configuration found that adding permeability heterogeneities tends to cause channelling; that is, flow predominantly along permeability layers rather than chaotic fingering (De Wit & Homsy 1997*b*; Sajjadi & Azaiez 2013; Shahnazari, Maleka Ashtiani & Saberi 2018). Loggia *et al*. (1996) found that when the injected fluid is more viscous than the ambient channelling is observed when the viscosity ratio is smaller than the ratio of permeabilities, and a single dispersive front is attained when the viscosity ratio is larger than the ratio of permeabilities. While these studies highlight some of the interesting qualitative behaviour that can be observed in miscible displacement flows when heterogeneity and viscosity variations interact, they do not provide a full overview of the different dynamical regimes that occur and the temporal evolution of the flow between them. The aim of this work is to identify the full lifecycle and evolution of stable and unstable miscible displacements in layered porous media, and to develop reduced-order models for the spreading and dispersion of the fluids, which can be used to quantitatively predict and up-scale flow in heterogeneous porous media.

This paper is laid out as follows. In § 2, we formulate the problem and outline the numerical method we use to solve it. In § 3, we consider neutrally stable displacements where the two fluids have the same viscosity. In § 4 we consider the effect of small viscosity variations, both stabilizing and de-stabilizing. In § 5 we consider large stabilizing viscosity variations and in § 6 we consider large de-stabilizing viscosity variations. In each of §§ 3–6 we discuss the time evolution of the concentration field and the different regimes through which the flow evolves, and derive reduced-order models for the evolution of the concentration field.

## 2 Problem formulation

**FIGURE 1:**
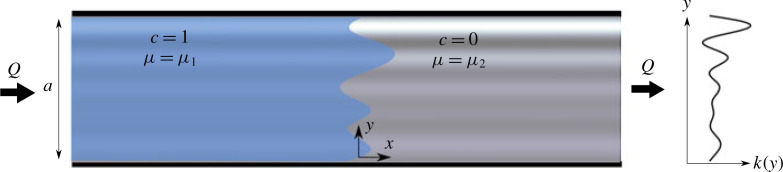
(Colour online) A schematic of the model geometry. The porous medium is semi-infinite with width *a* and has a permeability structure that is only a function of the transverse coordinate, *y*. The porous medium is initially saturated with a fluid of viscosity *μ*_2_ Another fluid, with viscosity *μ*_1_, which is fully miscible with the first, is injected at a constant flux *Q*.

A schematic of the problem geometry is given in figure 1. We consider a semi-infinite, two-dimensional porous strip with finite width *a*. For simplicity, the porosity of the medium, *φ*, is constant, but the permeability, *k* = *k(y)*, varies in the direction perpendicular to the flow. A fluid of viscosity *µ*_1_ is injected into the medium at a constant volumetric flux *Q*, which is initially saturated with an ambient fluid of viscosity *µ*_2_. The two fluids are fully miscible.

We assume that the flow obeys Darcy’s law everywhere, such that the pore-scale Reynolds number is small, the flow is incompressible, and the concentration, *c*, of the injected fluid evolves via advection and diffusion;

$$u=-\frac{k\left(y\right)}{\mu \left(c\right)}\nabla p,$$ (2.1)

∇ · u = 0, (2.2)

$$\phi \frac{\partial c}{\partial t}+u\;\cdot \;\nabla c=\phi D\nabla^{2}c.$$ (2.3)

Here ***u*** = (*u, v*) is the Darcy velocity and *p* is the pressure, and *D* is the effective diffusivity or dispersion coefficient between the two fluids, which in porous media tends to be velocity dependent, but is here assumed, for simplicity, to be a constant. The viscosity varies with the concentration of the injected fluid, and, following previous work (e.g. Tan & Homsy 1986), we assume an Arrhenius-like dependence,

$$\mu \left(c\right)=\mu_{2}e^{-Rc},$$ (2.4)

where *R* = log(*μ_2_/μ_1_*).

### 2.1 Non-dimensionalization

We non-dimensionalize the governing equations by the width of the porous medium *a*, the injection flux *Q*, the mean permeability $\bar{k}$, viscosity *μ*_2_, pressure $\mu_{2}Q/\bar{k}$ and convective time scale *φa*^2^/*Q*. We also change to a reference frame moving with the average speed of the injected fluid by setting $\tilde{x}=x-Qt/a$ and $\tilde{u}=u-Q/a$. Equations (2.1)–(2.4) become

$$-\left(\tilde{u}+1\right)\mu =k\frac{\partial p}{\partial \tilde{x}},\;-\nu \mu =k\frac{\partial p}{\partial y},$$ (2.5*a,b*)

$$\frac{\partial \tilde{u}}{\partial \tilde{x}}+\frac{\partial \nu }{\partial y}=0,$$ (2.6)

$$\frac{\partial c}{\partial t}+\tilde{u}\frac{\partial c}{\partial \tilde{x}}+\nu \frac{\partial c}{\partial y}=\frac{1}{pe}\left(\frac{\partial^{2}c}{\partial {\tilde{x}}^{2}}+\frac{\partial^{2}c}{\partial y^{2}}\right),$$ (2.7)

$$\mu \left(c\right)=e^{-Rc}.$$ (2.8)

The system is described by two non-dimensional parameters, the Péclet number *Pe* = *Q*/*D*, which characterizes the strength of advection to diffusion and the log-viscosity ratio *R*, as well as the non-dimensional permeability, *k*(*y*). For notational convenience we drop the tildes in all subsequent expressions.

We consider three different cases for the log-viscosity ratio: the neutrally stable case *R* = 0, where the injected and ambient fluids have the same viscosity; the ‘stable’ case *R* < 0, where the injected fluid is more viscous than the ambient; and the ‘unstable’ case *R* > 0, where the injected fluid is less viscous than the ambient. The Péclet number provides a measures of the relative strength of advection and diffusion, and in this work we will focus on the limit of large, but finite, Péclet number. This is the typical limit in most geologic scenarios.

### 2.2 Choice of permeability

In this paper, we consider only layered heterogeneous media for which *k* = *k(y)*. In fact, for all the numerical results presented here, we further restrict our attention to log-permeabilities which vary sinusoidally (De Wit & Homsy 1997*a*,*b*; Sajjadi & Azaiez 2013), such that

$$\ln\left(k\right)=-\sigma \;\cos\left(2\pi ny\right)-\ln{\mathrm{(I}}_{0}\mathrm{(\sigma ))},$$ (2.9)

where I_0_ is the modified Bessel function of the first kind, which ensures a unit average dimensionless permeability. This simplification retains the dominant physics of permeability heterogeneities in the form of layering, while being able to be described by two parameters instead of the infinite space of possible permeability functions. These two parameters are the log-permeability variance *σ* and wavenumber *n*, which measure the strength and inverse of the length scale of the permeability variations, respectively. While some of our results are presented for general *k*(*y*), all of the numerical simulations in this paper use this form for the permeability structure.

In the absence of hydrodynamic instabilities, as described in §§ 3–5, there is no mechanism for dynamic interactions between layers and so n can be scaled out of the system. This is done by introducing rescaled variables $\hat{y}=ny$, $\hat{x}=nx$, $\hat{t}=n^{2}t$, in which case the flow evolves exactly as it would with *n* = 1, but with an effective Péclet number $\hat{p}e=pe/n^{2}$. For clarity, we therefore limit our analysis in §§ 3–5 to the case where *n* = 1. If however the flow is unstable, as in § 6, there can be a competition between the evolving wavelength of the viscous fingering and the imposed wavelength of the permeability structure, resulting in rich intermediate-time dynamics for which the value of *n* can be important. We therefore explicitly consider the dependence of the flow on the number of layers in § 6.

### 2.3 Boundary and initial conditions

Following previous work (e.g. Tan & Homsy 1986; Nijjer *et al*. 2018), we consider flows that are periodic in the transverse (*y*) direction,

$$c\left(x,0,t\right)=c\left(x,1,t\right),$$ (2.10)

$$u\left(x,0,t\right)=u\left(x,1,t\right),\;\nu \left(x,0,t\right)=\nu \left(x,1,t\right).$$ (2.11*a,b*)

The upstream and downstream fluxes are zero (in the moving frame) and the transverse velocity vanishes in the far field so that,

$$\int_{0}^{1}u\;dy\to 0\;as\;x\to \pm \;\infty ,$$ (2.12)

$$\frac{\partial c}{\partial x}\to 0\;as\;x\to \pm \;\infty ,$$ (2.13)

ν→0 as x →±∞, (2.14)

We initialize the concentration field to have a step jump,

$$c\left(x,\;t=0\right)\;=c_{0}\left(x\right)=H\left(-x\right),$$ (2.15)

where *H*(*x*) is the Heaviside function.

### 2.4 Diagnostic quantities

In order to investigate how the macroscopic features of the flow evolve we focus, in the following analysis, on the evolution of the transversely averaged concentration $\bar{c}\left(x,t\right)=\int_{0}^{1}c\;dy$ and the mixing length *h*(*t*), defined below. To understand how $\bar{c}$ evolves, we start by decomposing the advection–diffusion equation (2.7), into two coupled equations for the transversely averaged concentration and the deviations, c'= $c-\bar{c}$

$$\frac{\partial \bar{c}}{\partial t}+\frac{\partial uc^{'}}{\partial x}=\frac{1}{pe}\;\frac{\partial^{2}\bar{c}}{\partial x^{2}},$$ (2.16)

and

$$\frac{\partial c'}{\partial t}+\frac{\partial uc'}{\partial x}+\frac{\partial \bar{uc}}{\partial x}-\frac{\partial \bar{uc^{'}}}{\partial x}+\frac{\partial \nu c'}{\partial y}=\frac{1}{pe}\left(\frac{\partial^{2}c^{'}}{\partial y^{2}}+\frac{\partial^{2}c^{'}}{\partial x^{2}}\right),$$ (2.17)

where $\bar{f}=\int_{0}^{1}f\;dy$ denotes the transverse average of the quantity *f* .These equations are obtained by averaging the advection–diffusion equation in the transverse direction and by subtracting the averaged equation from the unaveraged one. We return to this decomposition when describing the evolution of the transversely averaged concentration.

The region over which the two fluids have spread, the ‘mixing length’ *h*(*t*), is a measure of the streamwise extent of interpenetration of the two fluids. The mixing length provides a global measure of dispersion or spreading, and, while other quantities can give more direct measurements of the total amount of mixing (e.g. the scalar dissipation rate; Jha *et al*. 2011*a*), the mixing length provides a clear and physically useful measure of the region over which the two fluids have spread and are mixing. Here we define the mixing length to be the variance in concentration about the initial condition *c*_0_(*x*) (2.15),

$$h\left(t\right)=\sqrt{\frac{\int_{-\infty }^{\infty }x^{2}{\left(\bar{c}-c_{0}\right)}^{2}dx}{\int_{-\infty }^{\infty }{\left(\bar{c}-c_{0}\right)}^{2}dx}\cdot }$$ (2.18)

Note that we use this measure instead of the more common definition, h*= $x{|}_{\bar{C}=0.01}-\;x{|}_{0.99}$, because it more accurately captures the spreading behaviour when the concentration field has long tails (see appendix A).

Throughout this paper we make reference to the interface between the two fluids. Since the two fluids are fully miscible, there is no precise boundary. Instead, where we refer to the interface, we loosely mean the region around the *c* = 1/2 contour over which the concentration varies significantly.

### 2.5 Numerical method

We briefly summarize the numerical method here, but avoid a detailed exposition as the approach is very similar to that used by Nijjer *et al*. (2018). We start by combining (2.5), (2.6) and (2.8) and writing the velocity in terms of a streamfunction, $\left(u,v\right)=\left(\partial \Psi /\partial y,-\partial \Psi /\partial x\right)$, to give

$$\frac{\partial^{2}\Psi }{\partial x^{2}}+\frac{\partial^{2}\Psi }{\partial y^{2}}-\frac{\partial \Psi }{\partial x}\left(R\frac{\partial c}{\partial x}\right)-\frac{\partial \Psi }{\partial x}\left(R\frac{\partial c}{\partial y}+\frac{d\ln k}{dy}\right)=R\frac{\partial c}{\partial y}+\frac{d\ln k}{dy}\cdot$$ (2.19)

The transverse velocity boundary conditions (2.11) become

$$\Psi \left(x,0,t\right)=\Psi \left(x,1,t\right).$$ (2.20)

We imposed the boundary conditions in the flow direction (2.12)–(2.14), at a finite distance X=±Γ so that,

$$\frac{\partial \Psi }{\partial x}=0\;at\;x=\pm \Gamma ,$$ (2.21)

where *Γ* was chosen to be sufficiently large such that the mixing region was always far from the boundaries. In fact, we increased *Γ* over the course of each simulation so that it grew as the mixing length grew, ensuring that the mixing region was always far from the boundaries while the fine-scale dynamics at early times remained resolved. The domain was discretized on an adaptive rectangular grid which coarsened over time as the concentration gradients weakened.

The concentration field was initialized to an almost sharp interface, to avoid any numerical instabilities, by setting

$$c_{0}=\frac{1}{2}-\frac{1}{2}erf\left(x/\sqrt{t_{0}}\right)+r\left(x,y\right)e^{-x^{2}/t_{0}}.$$ (2.22)

Here *t*_0_ is a small time origin, which we set to *t*_0_ = 10^−6^ in all simulations, and *r* corresponds to a uniformly distributed random function, on the interval [0, 10^−4^], which was added to help trigger any instabilities.

At each time step (2.19) was solved using a multi-grid solver Adams (1999). Equation (2.3) was advanced in time using a third-order Runge–Kutta scheme. All spatial derivatives were discretized using sixth-order compact finite differences except for the advection term in (2.3), ***u*** **·** **∇***c*, which was discretized using a third-order upwinding scheme. We validated our scheme by comparison to the linear stability analysis of Pramanik & Mishra (2015*a*), and we chose spatial discretizations such that the smallest scales of the flow were always well resolved. In particular, we ensured that in cases when the interface was unstable to small-scale fingering the resolution was sufficiently high that doubling the grid spacing had no qualitative effect on the dynamics of the flow or diagnostic quantities.

## 3 Neutrally stable displacements *R* = 0

**FIGURE 2:**
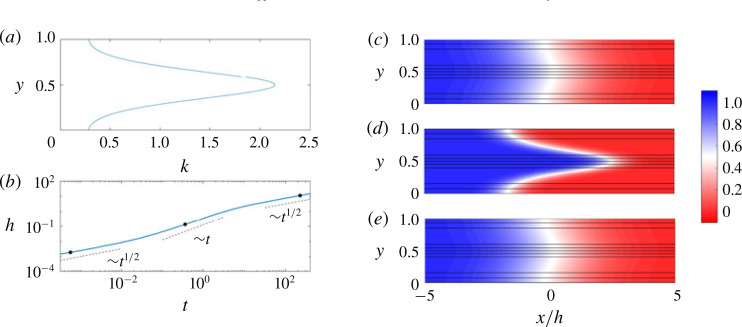
(Colour online) Evolution of the concentration field for *R* = 0 and (*σ*, *Pe*, *n*) = (1, 100, 1). (*a*) The imposed permeability *k*(*y*) = e^−cos(2πy)^/I_0_(1). (*b*) Evolution of the mixing length, *h*, as a function of time, *t*. The dots correspond to the snapshots (*c*–*e*). (*c*–*e*) Plots of the concentration field with overlain streamlines versus *x*/*h* and *y* at (*c*) *t* = 5 × 10−4, (*d*) *t* = 0.32 and (*e*) *t* = 200.

We first consider the case where the injected and ambient fluids have the same viscosity. While this case has been explored by a number of authors (e.g. Camacho 1993; Berentsen, Verlaan & van Kruijsdijk 2005; Dentz & Carrera 2007), we outline it here both for completeness and to set the stage for the analysis in the remainder of the manuscript. Results from a representative simulation are given in figure 2. As was noted earlier, if the flow is hydrodynamically stable, *n* can be scaled out of the problem and so we only consider the case where *n* = 1. In this case, the permeability is highest in the centre of the channel and lowest at the top and bottom boundaries (figure 2*a*). In the moving frame, this causes the fluid in the middle of the channel to move to the right and the fluid near the top and bottom boundaries to move to the left. This shear spreads and mixes the fluids. To quantify this spreading, we plot the evolution of the mixing length, *h*(*t*), in figure 2(*b*). We find that the mixing evolves through three distinct regimes each with a different scaling behaviour. The concentration fields corresponding to each of these regimes are plotted in figure 2(*c–e*). In the first and third regimes, the concentration fields look nearly indistinguishable: the concentration is nearly transversely uniform and relatively diffuse in the streamwise direction. In the second regime, there is a relatively sharp interface aligned with the permeability variations. Based on these observations, we expect that in the first (early-time) and third (late-time) regimes, the streamwise transport is diffusively dominated, whereas in the second (intermediate-time) regime, the streamwise transport is advectively dominated.

Since *R* = 0, the concentration acts as a passive tracer. This means that the velocity is decoupled from the concentration field, and is given from (2.5) by

$$u\left(y\right)=k\left(y\right)-1,\;\nu =0.$$ (3.1*a,b*)

In the case of sinusoidally varying log-permeability (2.9), the velocity is,

$$u=\frac{e^{-\sigma \cos\left(2\pi y\right)}}{I_{0}\left(\sigma \right)}-1,\;\nu =0.$$ (3.2*a,b*)

Given this fixed, known velocity the concentration simply evolves via the advection– diffusion equation (2.7). In the following sections, we consider the dominant balances in (2.7) to determine how the concentration field evolves in time.

### 3.1 Early-time behaviour: initial diffusion

At early times, the streamwise concentration gradient between the fluids is large and the concentration is transversely homogeneous. In this case, diffusion across the interface dominates and the primary balance in the advection–diffusion equation is

$$\frac{\partial c}{\partial t}=\frac{\partial \bar{c}}{\partial t}=\frac{1}{pe}\frac{\partial^{2}c}{\partial x^{2}}.$$ (3.3)

**FIGURE 3:**
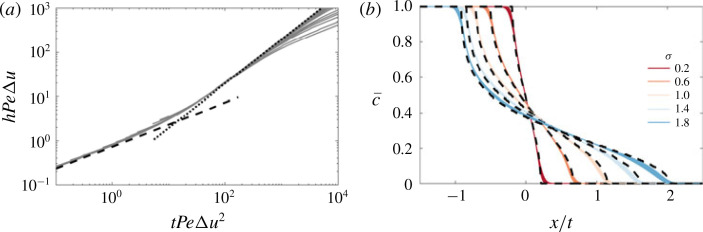
(Colour online) Evolution of the mixing length and transversely averaged concentration for *R* = 0 in the intermediate-time regime. (*a*) Scaled plot of the mixing length *h*(*t*) for (*Pe*, *σ* ) = (500, 1), *n* = {1, 2, 3, 4, 6, 8} and (*Pe*, *n*) = (500, 1), *σ* = {0.1, 0.2, 0.4, 0.6, 0.8, 1, 1.2, 1.4, 1.8, 2} and (*σ*, *n*) = (1, 1), *Pe* = {1, 2, 3, 4, 5, 6, 8, 15, 20} × 10^2^ . The black lines correspond to the predictions for the mixing length calculated from the analytical solutions (3.4) (dashed) and (3.7) (dotted). (*b*) Plot of the transversely averaged concentration, $\bar{C}\left(x,t\right)$ versus *x/t* for (*R, Pe, n*) = (0, 500, 1), *σ* ranging from 0.2 to 1.8 and ten logarithmically spaced times between 1 and 3. The characteristic spreading velocity is taken to be the maximum velocity difference between the layers, Δu ∼ sinh(σ )/I0 (σ ).

Using the initial and boundary conditions in § 2.3 the concentration evolves self-similarly as

$$c=\bar{c}=\frac{1}{2}+\frac{1}{2}erf\left(-\frac{x}{\sqrt{4t/pe}}\right),$$ (3.4)

which holds at all times when the permeability is homogeneous (*k* = 1). The mixing length grows like *h* ∼ *t*^1/2^ and can be calculated explicitly by substituting (3.4) into (2.18) (figure 3a).

This behaviour always holds initially, irrespective of the parameter choices, since diffusive growth of the interface *O*(*t^1/2^ /Pe*^1/2^) always outpaces advective spreading *O*(*Δut*) (where *Δu* is a characteristic spreading velocity). In fact, the transition to the intermediate regime occurs precisely when the growth rates become equal, giving a transition time *t* = *O*(1/*PeΔu*^2^ ).

### 3.2 Intermediate-time behaviour: advection

After a time *O*(1/*PeΔu*^2^), spreading induced by the difference in permeability overtakes longitudinal diffusion. The leading-order balance in (2.3) becomes

$$\frac{\partial c}{\partial t}+u\frac{\partial c}{\partial x}=0,$$ (3.5)

and the flow simply stretches the diffused solution that arises from the early-time regime. In fact, since the rate of advective stretching is much faster than diffusion to good approximation, we can ignore the effects of the early-time regime completely and the solution to (3.5) is simply the travelling wave

$$c=c_{0}\left(x-u\left(y\right)t\right)=H\left(u\left(y\right)-x/t\right),$$ (3.6)

given the initial condition (2.15). The transversely averaged concentration, $\bar{C}\left(x,t\right)$ can be calculated by averaging (3.6),

$$\bar{c}\left(x,t\right)=\int_{0}^{1}H\left(u\left(y\right)-x/t\right)dy.$$ (3.7)

**FIGURE 4:**
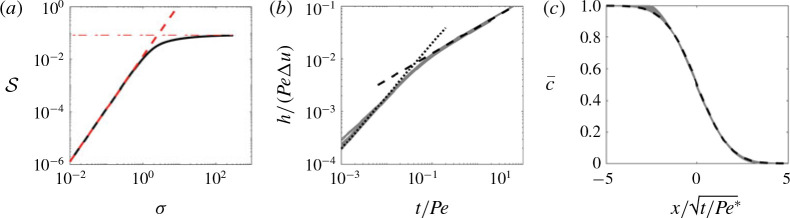
(Colour online) Evolution of the mixing length and transversely averaged concentration for *R* = 0 in the late-time regime. (*a*) Shear-enhanced dispersivity *S* versus *σ* with asymptotic limits (B 3) (dashed) and (B 6) (dot-dashed). (*b*) Scaled plot of *h* versus *t* for (*Pe, σ*) = (500, 1), *n* = {1, 2, 3, 4, 6, 8} and (*Pe, n*) = (500, 1), *σ* = {0.1, 0.2, 0.4, 0.6, 0.8, 1, 1.2, 1.4, 1.8, 2} and (*σ, n*) = (1, 1), *Pe* = {1, 2, 3, 4, 5, 6, 8, 15, 20} × 10^2^ . The black lines correspond to the predictions for the mixing length calculated from the analytical solutions (3.7) (dotted) and (3.4) with (3.11) (dashed). (*c*) Plot of the transversely averaged concentration, $\bar{c}\left(x,t\right)$ versus the late-time similarity variable $x/\sqrt{t/pe*}$ for the same parameters as (*b*) at *t* = 200. The theoretical solution (3.4) with (3.11) is given by the dashed line.

The model gives good agreement with the numerical simulations (figure 3*b*) and is able to reproduce the asymmetric profiles, which arise due to the fact that the permeability is not symmetric about *k* = 1 and collapses upon rescaling the streamwise coordinate by *t*. Since the interface is stretched at a constant rate, the mixing length grows like *h* ∼ *Δut* (figures 3*a* and 4*b*).

### 3.3 Late-time behaviour: shear-enhanced dispersion

The transition to the late-time regime occurs once the concentration has diffused across the entire channel, homogenizing the concentration in the transverse direction; this occurs at a time *O*(*Pe*). In this case, the mixing zone is long and thin and transverse diffusion balances longitudinal advection (cf. Taylor dispersion e.g. Taylor 1953; Aris 1956). This is in contrast to the previous regime, when the flow evolved purely by longitudinal advection. In the limit of small deviations from the mean $c'\ll \bar{c}$ and a long, thin mixing zone, (2.17) reduces to

$$\left(k-1\right)\frac{\partial \bar{c}}{\partial x}=\frac{1}{pe}\frac{\partial^{2}c'}{\partial y^{2}},$$ (3.8)

while the transversely averaged concentration still evolves according to (2.16). Given that $\bar{c}$ is independent of y, we integrate this equation twice and impose periodicity and zero-mean deviations $\left(\int_{0}^{1}c'=0\right)$ to give

$$c'=pe\frac{\partial \bar{c}}{\partial x}\left[\int_{0}^{y}\int_{0}^{\zeta }\left(k\left(\eta \right)-1\right)d\eta \;d\zeta -\int_{0}^{1}\int_{0}^{s}\int_{0}^{\zeta }\left(k\left(\eta \right)-1\right)d\eta \;d\zeta \;ds\right].$$ (3.9)

Substituting and solving for the convective flux in (2.16), using the expression for the velocity (3.1), leads to

$$\frac{\partial \bar{uc'}}{\partial x}=-pe\frac{\partial^{2}\bar{c}}{\partial x^{2}}\int_{0}^{1}{\left[\int_{0}^{y}\left(k\left(\eta \right)-1\right)d\eta \right]}^{2}\;dy.$$ (3.10)

This convective flux can be written in the form of an effective diffusivity such that (2.16) reduces to

$$\frac{\partial \bar{c}}{\partial t}=\frac{\partial }{\partial x}\left[\frac{1}{pe*}\frac{\partial \bar{c}}{\partial x}\right],\;\frac{1}{pe*}=\frac{1}{pe}\left(1+pe^{2}s\right);$$ (3.11*a,b*)

where

$$s=-\frac{1}{pe\partial \bar{c}/\partial x}\;\int_{0}^{1}uc'\;dy=\int_{0}^{1}{\left[\int_{0}^{y}\left(k\left(\eta \right)-1\right)d\eta \right]}^{2}dy$$ (3.12)

is the shear-enhanced dispersivity, which only depends on the permeability structure (cf. Van den Broeck & Mazo 1983).

For our choice of sinusoidally varying log-permeability, this integral cannot be solved analytically, but is instead integrated numerically for varying *σ* and plotted in figure 4(*a*). In appendix B, we derive the asymptotic limits of the shear-enhanced dispersivity for large and small *σ* (given as dashed and dot-dashed lines respectively in figure 4*a*).

The solution to (3.11*a*) is again the similarity solution (3.4), but now with a modified Péclet number *Pe∗* (3.11*b*) (see figure 4*c*). When the total dispersivity is dominated by the shear-enhanced dispersivity, *Pe*^2^*S* ≫ 1, the effective dispersion scales like *Pe*∗ ∼ 1/(*PeΔu*^2^). In figure 4(*b*) we use this scaling to collapse the mixing length as a function of time over a range of parameters.

In summary, in the presence of permeability layering but in the absence of viscosity variations, the flow evolves through three regimes: early-time diffusion, intermediate-time advection and late-time shear-enhanced dispersion.

## 4 Small viscosity variations |*R*| < *σ*

Next we consider the effect of viscosity variations that are weak compared to the permeability; that is, the log-viscosity ratio is smaller than the log-permeability variance, |*R*| < *σ*.

In the absence of permeability variations, when *R* > 0 and the Péclet number is sufficiently large, the flow is unstable and a set of complex nonlinearly evolving fingers develop (Tan & Homsy 1988). If permeability layering is introduced, the flow tends to be forced along the permeability pathways (De Wit & Homsy 1997*b*; Shahnazari *et al*. 2018) and as the permeability variance is increased, the flow becomes more and more channelized until the fingers no longer interact. This is especially true when the permeability variability dominates over variations in viscosity, *σ* ≫ |*R*|. Although instabilities are still possible (and are further discussed in § 6), in this section we focus on flows that remain hydrodynamically stable and follow the permeability pathways imposed.

**FIGURE 5:**
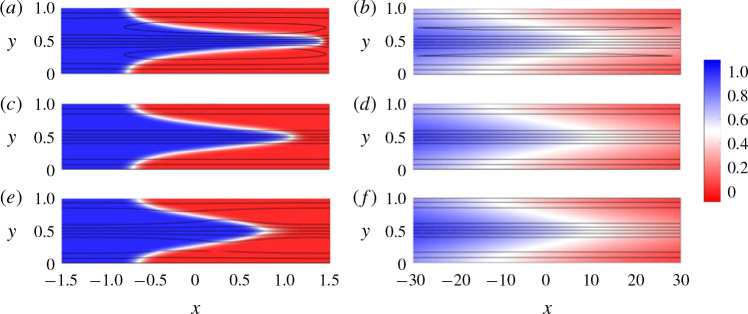
(Colour online) Colour maps of the concentration field with overlain streamlines for |*R*| < *σ*. (*a,b*) *R* = 0.4, (*c,d*) *R* = 0 and (*e,f*) *R* = −0.4 and (*σ, Pe, n*) = (1, 500, 1). The snapshots are taken at (*a,c,e*) intermediate times (*t* = 1) and (*b,d, f*) late times (*t* = 31). Note that the aspect ratios of the late-time figures are distorted.

Figure 5 shows the concentration field overlain with streamlines for *σ* = 1 and *R* = 0.4, 0 and −0.4 at intermediate times (left) and late times (right). For all three values of *R*, we find that the concentration field evolves in qualitatively the same manner: after an early-time diffusive regime, as in § 3.1, at intermediate times the flow is dominated by advective stretching (figure 5^a,c,e^); and at late times the flow is dominated by shear-enhanced dispersion (figure 5^b,d, f^ ). The main difference between flows where *R* ≠ 0 and *R* = 0 is that at intermediate times the interface is either stretched (*R* > 0) or compressed (*R* < 0) relative to the neutrally stable case owing to the viscosity-enhanced or viscosity-tempered streamwise velocity. At late times, the viscosity contrast seems to have little effect and the concentration field and streamlines look nearly indistinguishable. In the following subsections we examine the effects of small viscosity variations on the evolution of the three regimes identified in § 3. We begin in § 4.1 with a consideration of how viscosity contrasts affect the fluid velocity.

### 4.1 Vertical flow equilibrium

Unlike when the viscosities are equal, *R* = 0, we cannot simply integrate (2.5) to give a fixed expression for the velocity. As noted earlier, we find that when the injected fluid is more viscous than the ambient, *R* < 0, the streamwise velocity is reduced, whereas when the injected fluid is less viscous than the ambient, *R* > 0, the streamwise velocity is increased. Under the assumption that the flow is long and thin, the pressure is only a function of the longitudinal coordinate and is constant along any transverse slice to leading order. This limit, often referred to as ‘vertical flow equilibrium’ (Yortsos 1995), implies that

$$\frac{\mu \left(u+1\right)}{k}=-\frac{dp}{dx}\approx const.$$ (4.1)

Combining (4.1) with the fact that the flux vanishes in any transverse slice in the moving frame, the velocity can be written as

$$u\left(x,y,t\right)=\frac{k\left(y\right)}{\mu \left(x,y,t\right)}{\left[\int_{0}^{1}\frac{k\left(s\right)}{\mu \left(x,y,t\right)}ds\right]}^{-1}-1.$$ (4.2)

If the viscosity is uniform, then the permeability sets the velocity, *u* = *k* − 1, as in (3.1). If, instead, the permeability is uniform, then the viscosity sets the velocity, $u\left(y\right)=\mu^{-1}/\int_{0}^{1}\mu^{-1}dy-1$ (this leads to the fast low-viscosity fingers and slow high-viscosity fingers characteristic of the viscous-fingering instability). When both the permeability and viscosity vary, depending on the sign of *R* and *c′*, the permeability and viscosity can interact either constructively or destructively. The effect of varying viscosity is only important at the interface; far upstream and downstream, where the viscosity is uniform, the velocity variations are simply imposed by the structure of the permeability field.

Decomposing the concentration into the transverse average and deviations c= $\bar{c}\left(x\right)+c'\left(x,y\right)$, equation (4.2) becomes independent of the average concentration and only depends on the transverse variations,

$$u\left(x,y,t\right)=\frac{k\left(y\right)}{e^{-Rc'\left(x,y,t\right)}}{\left[\int_{0}^{1}\frac{k\left(s\right)}{e^{-Rc'\left(x,s,t\right)}}ds\right]}^{-1}-1.$$ (4.3)

In the case of sinusoidal log-permeability variations, equation (4.3) is

$$u\left(x,y,t\right)=\frac{e^{-\sigma \cos\left(2\pi y\right)+Rc'\left(x,y,t\right)}}{\int_{0}^{1}e^{-\sigma \cos\left(2\pi ys\right)+Rc'\left(x,s,t\right)}ds}-1.$$ (4.4)

### 4.2 Intermediate-time behaviour: viscously coupled advection

**FIGURE 6:**
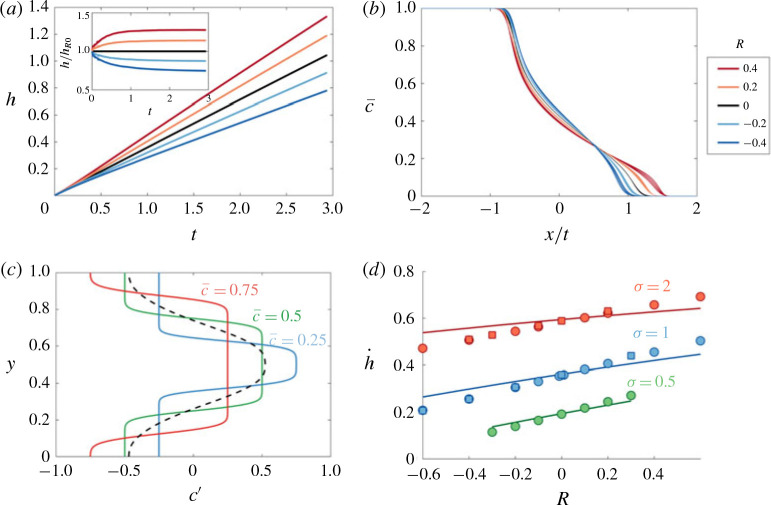
(Colour online) Evolution of the mixing length and transversely averaged concentration for |*R*| < *σ* in the intermediate-time regime. (*a*) Evolution of the mixing length for (*σ , Pe, n*) = (1, 2000, 1) and *R* ranging from −0.4 to 0.4; the inset shows the same data normalized by the neutral displacement mixing length. (*b*) Plot of $\bar{c}$ versus *x/t* for the same parameters as (*a*) for 10 logarithmically spaced times in the range 1 ≤ *t* ≤ 3. (*c*) Plot of the concentration deviations *c*′ as a function *y* at three different points in *x* corresponding to $\bar{c}=0.5$ (red), $\bar{c}=0.25$ (green) and $\bar{c}=0.75$ (blue) for (*R, σ, Pe, n*) = (−0.4, 1, 2000, 1). The longitudinally averaged concentration deviations (averaged over the mixing zone), $\int_{h}c^{\prime }dx/h$, is given by the dashed black line. (*d*) Plot of the spreading rate $\dot{h}$ calculated by least-squares fitting a function of the form $h=h_{0}+\dot{h}t$ to the numerical results for t in the range 1 ≪ *t* ≪ 3, for *Pe* = 2000 (circles) and *Pe* = 4000 (squares). The theoretical predictions for $\dot{h}$ calculated using (4.5) and (3.7), are given by the solid lines.

After the early-time diffusion regime the flow transitions to the intermediate-time regime dominated by advective spreading. The effect of a non-zero viscosity ratio on the evolution of the mixing length is shown in figure 6(*a*). Similar to the *R* = 0 case, the mixing zone grows linearly in time, however as *R* is increased, the growth rate of the mixing zone, $\dot{h}=dh/dt$, increases. The nearly uniform spacing between the curves suggests that the growth rate varies linearly in *R*. The transversely averaged concentration is again asymmetric and evolves self-similarly (figure 6*b*), although it differs appreciably from the *R* = 0 case at the downstream tips (cf. snapshots in figure 5*a,c,e*).

In this regime, we first note that diffusion is negligible. This results in concentration deviations that are almost exactly either $c^{\prime }=-\bar{c}$ or $c^{\prime }=1-\bar{c}$ (figure 6*c*). To estimate the overall effect of the deviations on the velocity, we average the deviations across the length of the fingered region, which leads to a roughly sinusoidal variation across the domain aligned with the permeability structure and with magnitude ≃= 1/2 (dashed black line in figure 6*c*). Substituting these average deviations into (4.4), the mean streamwise velocity reduces to

$$u=\frac{e^{-\left(\sigma +R/2\right)\cos\left(2\pi y\right)}}{\int_{0}^{1}e^{-\left(\sigma +R/2\right)\cos\left(2\pi s\right)}ds}-1.$$ (4.5)

This approximate model results in intermediate-time dynamics equivalent to the neutrally stable case, but with an effective log-permeability ratio *σ_eff_ = σ + R/2*. Given (4.5), we can calculate *h* as in § 3.2, and extract $\dot{h}$ by fitting a linear profile $h=\dot{h}t$. Modelling the effective permeability in this way gives reasonably good agreement with the numerical simulations (figure 6*d*), although it underestimates the spreading rate for large |*R*|. This is because, at the boundary of the forward-propagating and backward-propagating tips, the velocity is faster, *u = e^σ+R^/I_0_*(*σ*), and slower, *u = e^−(σ +R)^/I_0_*(*σ*), respectively, than this model predicts.

### 4.3 Late-time behaviour: viscosity-dependent shear-enhanced dispersion

At late times, after advectively spreading, the concentration evolves diffusively again. This evolution is analogous to the late-time behaviour of the neutrally stable case but the addition of viscosity variations modifies the effective diffusivity.

As in § 3.3, we assume that the flow is long and thin, transverse velocities are negligible so the fluid flow is predominantly in the streamwise direction and the concentration deviations are small and evolve on a much faster time scale than their transverse average. Equation (2.16) remains unchanged, but (3.8) becomes

$$\left(\frac{ke^{Rc'\left(x,y,t\right)}}{\int_{0}^{1}ke^{Rc'\left(x,s.t\right)}ds}-1\right)\frac{\partial \bar{c}}{\partial x}pe=\frac{\partial^{2}c'}{\partial y^{2}}\left(x,y,t\right)$$ (4.6)

because of the dependence of the velocity on the concentration through (4.3). Again we impose periodic boundary conditions and zero-mean deviations.

**FIGURE 7:**
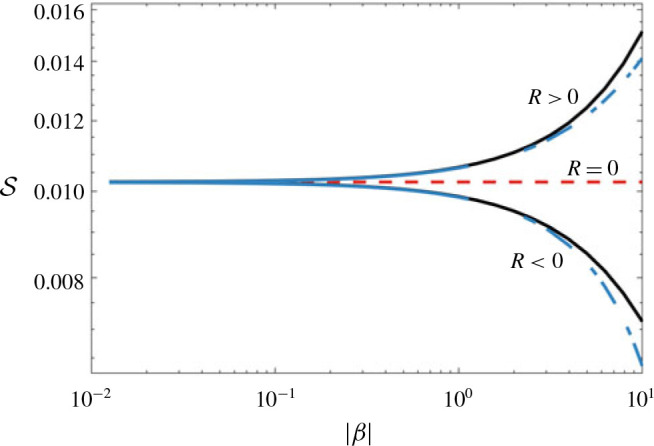
(Colour online) Plot of *S* versus |*β*| for *R* < 0 and *R* > 0. The solid black lines correspond to *S* , equation (4.8), calculated by numerically integrating (4.7). The leading-order small-|*β*| asymptotic behaviour, which is equal to when *R* = 0, is given by the dashed red line and the next-order corrections in *β* are given by the dot-dashed blue lines, equation (4.12).

Before solving, we first rescale the deviations, $\tilde{c}=Rc^{\prime }$, such that (4.6) becomes

$$\left(\frac{ke^{\tilde{c}}}{\int_{0}^{1}ke^{\tilde{c}}ds}-1\right)\;\beta =\frac{\partial^{2}\tilde{c}}{\partial y^{2}},$$ (4.7)

where $\beta =R\;pe\;\partial \bar{c}/\partial x=pe\;\partial$ ln $\mu \left(\bar{c}\right)/\partial x$, incorporates all of the parameters in the problem, and can be thought of as a rescaled bulk concentration or viscosity gradient. Since *β* is independent of *y*, we can solve for $\tilde{c}$ by integrating (4.7) twice. We perform this integration numerically, although the limits of small *β*, which is relevant here and is considered in § 4.3.1, and large *β*, which we find to be useful and is considered later in § 5.1, can be treated analytically. Having solved for $\tilde{c}$, we then calculate the shear-enhanced dispersivity,

$$s=-\frac{1}{\beta }\int_{0}^{1}u\tilde{c}\;dy,$$ (4.8)

(cf. (3.12)). Solutions of *S* for *σ* = 1 and *R* > 0 and *R* < 0 are given in figure 7. When *R* = 0, (4.7) reduces to (3.8) and *S* is exactly as described by (3.12), a permeability-dependent constant. When the viscosity varies, the dispersivity is enhanced when the injected fluid is less viscous (*R* > 0) and diminished when the injected fluid is more viscous (*R* < 0), than the ambient fluid. Since $\beta \;\propto \;\partial \bar{c}/\partial x$, and diffusion always causes concentration gradients to diminish in time, *β* generally decreases over time. When |*β*| is large (i.e. the viscosity gradient is large, as at early times), and *R* > 0, *S* diverges, whereas when *R* < 0, *S* tends to zero. The former limit is unphysical and corresponds to scenarios where the interface is unstable. The latter case is discussed in more detail in § 5, in the context of stable injections. When |β | is small (i.e. the viscosity gradient is small, as at late times), *S* becomes independent of *β* and tends to the value for *R* = 0. This suggests that at late enough times, the effective diffusivity will always become independent of the log-viscosity ratio, and the flow will always evolve like the neutrally stable case.

When |*R*| < *σ* , we need to only consider the small-|*β*| limit because by the time the flow reaches the late-time regime, *β* is inevitably small. We can justify this claim using a scaling argument: once the flow reaches the late-time regime, which occurs at a time *t = O(Pe)*, the mixing length will have grown to a width *h = O(PeΔu)*, or equivalently, the concentration gradient is $\partial \bar{c}/\partial x=O\left(1/pe\Delta u\right)$. This means that when the flow transitions to the late-time regime, |*β*| = *O(R/Δu) < O(1)*.

#### 4.3.1 Small viscosity gradient limit: |*β*| 1

For *β* ≪ 1, we start by expanding the concentration deviations as $\tilde{c}=\beta {\tilde{c}}_{1}+\beta^{2}{\tilde{c}}_{2}+$ $O\left(\beta^{3}\right)$. Substituting into (4.6), expanding and equating powers of *β* gives

$$\frac{\partial^{2}{\tilde{c}}_{1}}{\partial y^{2}}=k-1\;,\;\frac{\partial^{2}{\tilde{c}}_{2}}{\partial y^{2}}=\left(k{\tilde{c}}_{1}-k\bar{k{\tilde{c}}_{1}}\right).$$ (4.9*a,b*)

Given that the concentration deviations must satisfy periodicity and have vanishing mean, we find that

$${\tilde{c}}_{2}=I\left(k-1\right)-\bar{I}\left(k-1\right),$$ (4.10)

$${\tilde{c}}_{2}=I\left(k{\tilde{c}}_{1}\right)-I\left(k{\tilde{c}}_{1}\right)-\bar{k{\tilde{c}}_{1}}I\left(k\right)+\bar{k{\tilde{c}}_{1}}\bar{I}\left(k\right),$$ (4.11)

where $I\left(f\right)=\int_{0}^{y}\int_{0}^{\zeta }f\;d\eta \;d\zeta$ for a given function *f* and, as before, the overbar refers to a transverse average. The shear-enhanced dispersivity, *S* , is thus

$$s=-\frac{1}{\beta }\int_{0}^{1}u\tilde{c}\;dy=\bar{k{\tilde{c}}_{1}}+\beta \bar{(k{\tilde{c}}_{1}^{2}}-{\bar{k{\tilde{c}}_{1}}}^{2}+\bar{k{\tilde{c}}_{2})}+o(\beta^{2}).$$ (4.12)

The leading-order contribution to *S* is identical to the *R* = 0 limit in § 3.3 and is plotted in figure 7 as a dashed red line. The first-order corrections are given by dot-dashed blue lines.

### 4.3.2 Comparison to numerical simulations

We now use the calculated shear-enhanced dispersivity to determine how $\bar{c}$ evolves in time. Allowing *S* to vary in space, (2.16) yields a nonlinear diffusion equation for $\bar{c}$,

$$\frac{\partial \bar{c}}{\partial t}=\frac{1}{pe}\frac{\partial }{\partial x}\left\{\left[1+pe^{2}s\left(\beta \left(x\right)\right)\right]\frac{\partial \bar{c}}{\partial x}\right\},$$ (4.13)

where $\beta \left(x\right)=R\;pe\;\partial \bar{c}/\partial x$ depends on the local mean concentration gradient. We solve (4.13) numerically with no-flux boundary conditions in the far field using a Crank–Nicolson predictor–corrector method. We use both the exact form for *S* (by solving (4.7) and calculating (4.8)), and the small-|*β*| approximation in (4.12). The model concentration fields are initialized with a diffuse error-function solution although the long-time results are indifferent to the exact initial conditions. The non-uniform dispersion results in profiles that deviate from the classical error-function solution owing to the enhanced or diminished dispersion in regions of large concentration gradients.

**FIGURE 8:**
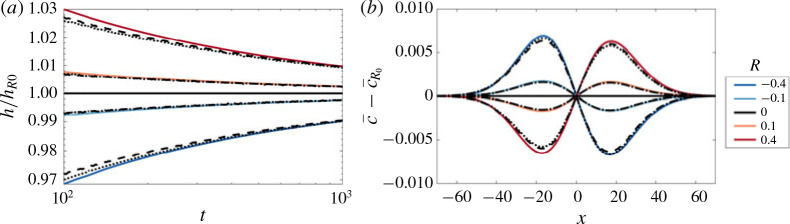
(Colour online) Evolution of the mixing length and transversely averaged concentration for |*R*| < *σ* in the late-time regime. (*a*) Evolution of the mixing length *h*, normalized by the neutral displacement mixing length *h_R=0_* for (*σ, Pe, n*) = (1, 100, 1) and *R* ranging from –0.4 to 0.4. (*b*) Plot of the difference between $\bar{c}$ for *R* = 0 and *R* = 0. The profiles are measured at *t* = 100 for the same parameters as (*a*). The theoretical predictions, found by solving (4.13) with either the exact solution of *S* (by solving (4.7)), or (4.12), are given by the dashed and dotted black lines respectively.

Figure 8(*a*) shows the evolution of the normalized mixing length, *h/h_R_0__*, where *h_R_0__ = h(t, R* = 0), for different *R*, from the full two-dimensional (2-D) numerical simulations (solid coloured lines) and model results (dashed and dotted black lines). As expected, increasing *R* results in increased spreading, while the effects of variations in the viscosity reduce as *t* is increased. The reduced-order model not only captures this behaviour but also accurately predicts the manner in which the flow evolves. This can be further seen in figure 8(*b*) which compares the reduced-order model predictions for $\bar{c}$ with the full 2-D numerical simulations. The very good agreement between both of the model solutions and the numerical results suggests that the late-time behaviour can be accurately modelled by (4.13) with (4.12).

## 5 Large stabilizing viscosity variations |*R*| > σ , *R* < 0

Whereas in the previous section the flow evolves in qualitatively the same manner as the uniform viscosity case (*R* = 0), when the viscosity ratio is larger than the permeability ratio, the concentration evolves in a qualitatively different manner. Here we consider the case where the injected fluid is more viscous than the ambient fluid (*R* < 0) and the magnitude of the log-viscosity ratio is larger than the log-permeability ratio.

**FIGURE 9:**
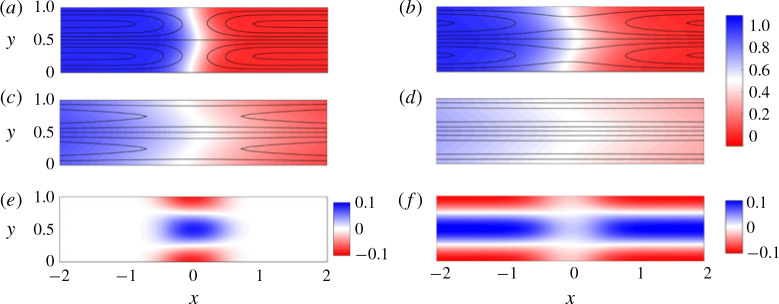
(Colour online) Evolution of the concentration field for stable displacements |*R*| > σ, *R* < 0. (*R*, σ, *Pe*, *n*) = (−1, 0.1, 100, 1). (*a–d*) Colour maps of the concentration field with overlain streamlines at (*a*) *t* = 3.6, (*b*) *t* = 17.8, (*c*) *t* = 89.1 and (*d*) *t* = 447. (*e*) Colour map of the concentration deviations $c^{\prime }=c-\bar{c}$ at *t* = 3.6. (*f*) Colour map of the streamwise velocity *u* at *t* = 3.6.

Figure 9(*a–d*) shows a sequence of snapshots of the concentration field in this limit. The interface is initially not stretched by the permeability variations owing to the large longitudinal gradient in viscosity (figure 9a). This is because, for large |*∂c/∂x*|, distorting the interface generates large transverse gradients in concentration. These correspond to large, stable transverse gradients in viscosity (and hence pressure) which tend to force the profile back to vertical. Ultimately, this results in a stationary interface (in the moving frame) where the streamwise velocity goes to zero (figure 9f ). Far upstream and downstream, where the viscosity is transversely uniform, the velocity is imposed by the permeability. The abrupt change in velocity at the interface drives a circulation on either side of the interface carrying concentration away from it. As the fluids mix, the concentration, and hence viscosity gradients at the interface weaken. Over time, the stabilizing viscous forces become sufficiently weak that the streamwise velocity can grow and streamlines begin to penetrate through the interface (figure 9b,c). Eventually the interface becomes very diffuse and the velocity becomes predominantly longitudinal (figure 9d) as in the cases considered in previous sections. In contrast to those cases, however, where the concentration deviations, (*c*′), are *O*(1) until the late-time regime, the concentration deviations here are always small because the concentration remains relatively transversely uniform with no large-scale channelling into layers.

### 5.1 Concentration model

As found in § 4.3, the shear-enhanced dispersivity *S* is given by (4.8) where *β* = $R\;pe\;\partial \bar{c}/\partial x$ and $\tilde{c}$ is given by (4.7) and *u* by (4.3). However, unlike in § 4.3, for viscosity-stabilized flows, we now expect this model to apply for all time, rather than just at late times, given that the flow remains nearly transversely uniform (*c*′ ≪ 1). In particular, |*β*| is not just small but can take on any value. We thus first consider the large-*β* limit.

We start by expanding $\tilde{c}$ in powers of $1/\beta ,\;\tilde{c}={\tilde{c}}_{0}+{\tilde{c}}_{1}/\beta +O\left(1/\beta^{2}\right)$. Substituting into (4.7) expanding and equating different powers of *β*, we find

$$\frac{ke^{{\bar{c}}_{0}}}{\int_{0}^{1}ke^{{\bar{c}}_{0}}\;dy}-1=0,$$ (5.1)

and so,

$${\tilde{c}}_{0}=-\ln\left(k\right)+\bar{\ln\left(k\right),}\;{\tilde{c}}_{0}=\frac{\partial^{2}{\tilde{c}}_{0}}{\partial y^{2}},$$ (5.2*a,b*)

and

$${\tilde{c}}_{1}=-\left(\frac{kk^{\prime \prime }-{\left(k^{\prime }\right)}^{2}}{k^{2}}\right),$$ (5.3)

where we have again used the fact that $\int_{0}^{1}{\tilde{c}}_{0}=\int_{0}^{1}{\tilde{c}}_{1}=0$. To leading order, the concentration deviations align themselves such that the streamwise velocity is zero. The shear-enhanced dispersivity in this limit is

$$S=\frac{1}{\beta }\int_{0}^{1}u\tilde{c}\;dy=\frac{1}{\beta^{2}}\int_{0}^{1}\ln\left(k\right)\left(\frac{kk^{\prime \prime }-{\left(k^{\prime }\right)}^{2}}{k^{2}}\right)\;dy+0\left(\frac{1}{\beta^{3}}\right).$$ (5.4)

In the case of sinusoidally varying log-permeability, this limit corresponds to

$$S=\frac{2\sigma^{2}\pi^{2}}{\beta^{2}}+0\left(\frac{1}{\beta^{3}}\right).$$ (5.5)

**FIGURE 10:**
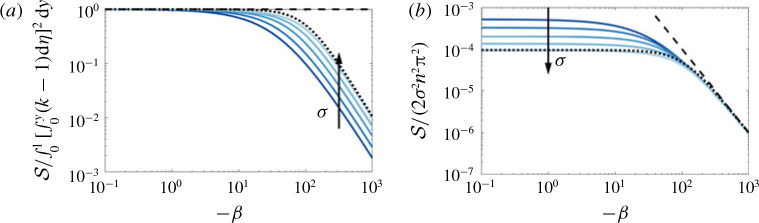
(Colour online) (*a*) Plot of *S* normalized by its small-|*β*| limit versus *β* and (*b*) plot of *S* normalized by its large-|*β*| limit versus *β* for σ ranging from 1 to 5. The dashed black lines denote the asymptotic limits (4.12) and (5.4) in (*a*) and (*b*) respectively and the dotted lines correspond to the approximate solution (5.6) for *σ* = 5.

Equations (4.12) and (5.4) correspond to the asymptotic limits of small and large viscosity gradient, and are plotted together with the full solutions of (4.7)–(4.8) for the shear-enhanced dispersivity in figures 10(*a*) and 10(*b*) respectively. We can also combine the two limits into a very simple approximate analytical composite solution; for example for the case of a sinusoidally varying log-permeability,

$$S_{comp}={\left(\frac{1}{S^{*}}+\frac{\beta^{2}}{2\sigma^{2}\pi^{2}}\right)}^{-1},$$ (5.6)

where $S*={\int_{0}^{1}\left[\int_{0}^{y}\left(k-1\right)\;d\eta \right]}^{2}dy$ is the leading-order behaviour in (4.12). This approximate solution captures the general behaviour of the shear-enhanced dispersivity reasonably well without having to solve (4.7) exactly (see dotted lines in figure 10).

### 5.2 Comparison to numerical simulations

**FIGURE 11:**
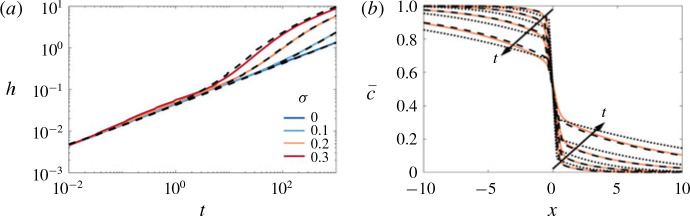
(Colour online) Evolution of the mixing length and transversely averaged concentration for |*R*| > *σ*, *R* < 0. (*a*) Plot of *h* versus *t* for (*R*, *Pe*, *n*) = (−2, 300, 1) and *σ* ranging from 0 to 0.3. (*b*) Plot of $\bar{c}\left(x,t\right)$ for (*R*, σ , *Pe*, *n*) = (−2, 0.2, 300, 1) and t = 32, 100, 320, 1000. The theoretical predictions, found by solving (4.13), with either the exact solution of *S* (found by solving (4.7)), or (5.6), are given by the dashed and dot-dashed black lines respectively.

We solve the reduced-order model (4.13) both directly and using the approximate composite solution (5.6), in the same manner as § 4.3.2 with a step initial condition (2.15). The comparisons to the transversely averaged concentration profiles from the full numerical simulations are given in figure 11(*b*). The exact solution of the reduced model is not only able to capture the sharp interface and the long tails, but it also accurately predicts the evolution of the concentration field. Using the composite approximation of *S* in the reduced model also captures the qualitative evolution of the transversely averaged concentration although it slightly overestimates the amount of spreading that occurs. The mixing length is also shown as a function of time for different σ in figure 11(*a*). The very good agreement between the reduced-order model and the full simulations over a range of *σ* and *t*, suggests that the full 2-D problem can be reduced to solving a 1-D nonlinear diffusion equation for all times in this limit.

## 6 Large de-stabilizing viscosity variations |*R*| > *σ*, *R* > 0

Finally, we consider the case where the injected fluid is less viscous than the ambient fluid and the log-viscosity ratio is larger than the log-permeability ratio, *R* > σ . In the absence of any permeability variations, *σ* = 0, this configuration is hydrodynamically unstable (provided Pe is sufficiently large). In homogeneous media such unstable miscible displacements evolve through three flow regimes (Nijjer *et al*. 2018): at early times, the flow is linearly unstable, where the interface grows diffusively, and fingers grow exponentially; at intermediate times, the fingers have finite amplitude and propagate and interact with each other nonlinearly leading to coarsening; and at late times, a single pair of counter-propagating fingers remain which propagate and slow, leaving a well-mixed interior.

In the presence of permeability layering, there is a competition between the evolving wavelength of viscous fingering and the imposed wavelength of the permeability structure. The competition between viscous fingering, which acts to coarsen the transverse length scale, and permeability layering, which acts to impose a fixed length scale, results in rich intermediate-time dynamics. As a result, the number of layers *n* can no longer be scaled out of the problem. Nonetheless, as before, the early-time dynamics is still dominated by longitudinal diffusion across the sharp interface, and the late-time dynamics is dominated by shear-enhanced dispersion which becomes independent of the viscosity ratio at long times (cf. § 4.3).

**FIGURE 12:**
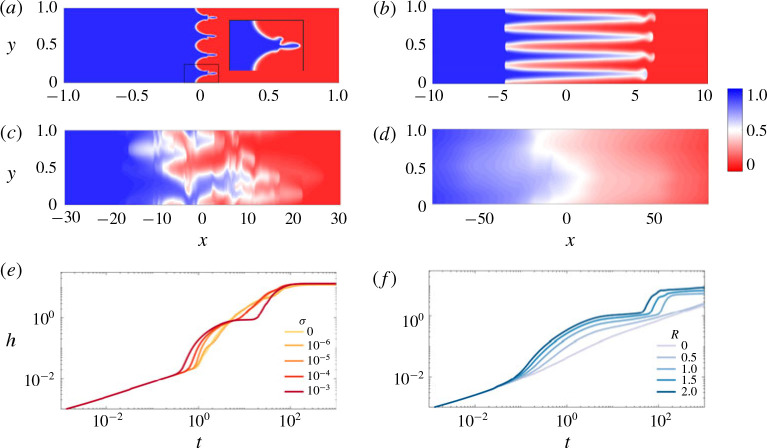
(Colour online) Evolution of the concentration field for |*R*| > σ and *R* > 0. (*a–d*) Colour maps of the concentration field for (*R*, σ , *Pe*, *n*) = (2, 0.1, 4000, 4) at (*a*) *t* = 0.1, (*b*) *t* = 8, (*c*) *t* = 104 and (*d*) *t* = 1500. Note that the aspect ratio is increasingly compressed in figures (*b–d*). (*e, f*) Evolution of *h*(*t*) for (*Pe*, *n*) = (1000, 4) and: (*e*) *R* = 2 with varying *σ*; and (*f*) σ = 0.1 with varying *R*.

In general, there are four possible intermediate-time regimes through which the flow can evolve, representative snapshots of which are given in figure 12. In the first regime (I), fingering occurs within the permeability layers and these fingers coarsen until they coincide with the imposed permeability layering (figure 12*a*). In the secondregime (II), the flow follows the imposed layered structure while diffusing across the layers causing the flow to slow down (figure 12*b*). In some cases, this flow can then be unstable, leading to a third regime (III) which corresponds to fingering over a transverse length scale that is larger than that imposed by the permeability (figure 12*c*). These fingers then also coarsen, leading to a fourth regime (IV) where a single pair of counter-propagating fingers remain (figure 12*d*). This pair of fingers slows leaving a well-mixed region which eventually evolves through shear-enhanced dispersion. Note that regimes III and IV can only occur if *n* > 1.

The growth of the mixing length can be drastically different depending on which regime the flow is in (figure 12*e,f*). When the flow fingers (regimes I and III), as with a homogeneous porous medium, the mixing length grows linearly in time. When the flow is channelling or in the single-finger exchange-flow regime (regimes II and IV) the mixing length tends to a constant value. As *σ* is varied from 0 (the homogeneous medium scenario), we see a transition from pure viscous fingering to channelling and fingering behaviour (figure 12*e*). As *R* is varied from 0 (neutrally stable displacement scenario), we see a transition from pure channelling behaviour to both channelling and fingering (figure 12*f*). Exactly which regimes occur depend on all four of the variables in this problem. In general we find fingering behaviour (regimes I and III) is more significant for larger values of *R* and *Pe*, while channelling is more significant for larger values of *σ*. Also, fingering across layers (regimes III and IV) is only relevant when *n* > 1, and is most notable when the length scale of the permeability variations is small, *n* ≪ 1.

In the following sections we review the different regimes briefly and discuss some simple models for the evolution of the transversely averaged concentration. We note that the following is not an exhaustive description of all possible behaviour, and we leave an exhaustive description of the interplay between viscous fingering and permeability layering for future work.

### 6.1 Regime I: fingering within layers

The flow is able to finger within the permeability layers when the length scale of the most unstable mode, *y* ~ 1/*RPe* (Tan & Homsy 1986), is small compared to the length scale imposed by the permeability *y* ~ 1/*n*. Hence the flow always fingers for sufficiently large Pe and is also observed to be enhanced when *σ* is small (De Wit & Homsy 1997b; Shahnazari *et al*. 2018). Note that fingering within layers is also possible when permeability effects dominate over viscous effects, *R* < *σ*, so long as *Pe* is sufficiently large.

**FIGURE 13:**
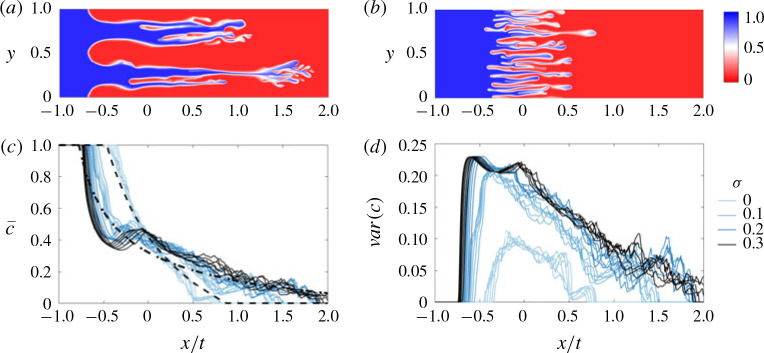
(Colour online) Evolution of the concentration field for |*R*| > *σ* and *R* > 0 in the fingering within layers regime; (*R*, *Pe*, *n*) = (2, 8000, 2). (*a,b*) Colour maps of the concentration field for (*a*) σ = 0.1 and (*b*) *σ* = 0 at *t* = 1. (*c*) Plot of the transversely averaged concentration $\bar{c}\left(x,t\right)$ and (*d*) transverse variance in concentration $\mathrm{var}\left(c\right)=\int_{0}^{1}{c^{\prime }}^{2}dy$ versus the similarity variable *x/t* for *σ* ranging from 0 to 0.3. In (*c*), the theoretical prediction for a homogeneous medium (Nijjer *et al*. 2018, dashed) and for a heterogeneous medium ((C 4), dot-dashed) are also shown.

Figure 13(*a,b*) shows snapshots of the concentration field for *σ* = 0.1 and *σ* = 0 respectively. The fingers evolve in a similar manner in both cases, leading to an asymmetric transversely averaged concentration that evolves self-similarly with similarity variable *x/t* (figure 13c). The main difference between the two simulations is that the fingers move significantly faster when permeability heterogeneities are present. For example, the mixing length grows at more than double the rate in the simulation in figure 13(*a*) compared to the homogeneous case, which is far larger than one would predict simply by considering the difference in permeability.

This difference is due to the structure of the flow being fundamentally different in the two simulations. Whereas, in the homogeneous medium, the forward and backward propagating fingers are on average about the same size, in the heterogeneous medium, the backward propagating fingers are broad and aligned with the low permeability layers. This means the two fluids tend to be much more segregated (figure 13*d*), and so the effective viscosity difference between the forward- and backward-propagating fingers is much larger in the heterogeneous medium, which leads to much faster spreading. We also observe from the simulations that the spreading rate is broadly insensitive to *σ* for *σ* ≳ 0.1 (figure 13*c,d*), which supports the idea that the presence of layered heterogeneity changes the spreading rate through the structure of the flow. In § C.1 we extend the model presented by Nijjer *et al*. (2018) for the homogeneous case by accounting for this change in finger structure. The model prediction, given by the dot-dashed line in figure 13(*c*), gives good quantitative agreement with the numerical simulations when *σ* ≳ 0.1. Thus we find that, although small amounts of permeability heterogeneity would be expected to have a small effect on the spreading rate, they can in fact alter the structure of the flow and lead to significantly faster spreading and mixing.

### 6.2 Regime II: stable channelling

**FIGURE 14:**
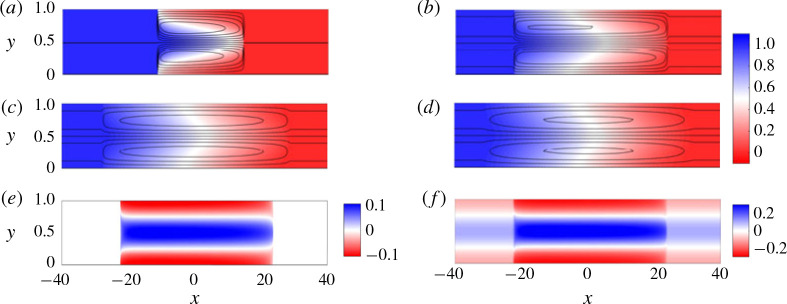
(Colour online) Evolution of the concentration field for |*R*| > *σ* and *R* > 0 in the channelling regime; (*R*, σ, *Pe*, *n*) = (2, 0.1, 500, 1). (*a–d*) Colour maps of the concentration field with overlain streamlines at (*a*) *t* = 20, (*b*) *t* = 60, (*c*) *t* = 100 and (*d*) *t* = 140. (*e*) Colour map of the concentration deviations $c^{\prime }=c-\bar{c}$ at *t* = 20. (*f*) Colour map of the streamwise velocity *u* at *t* = 20. Note that the aspect ratio of the figures is compressed by a factor of 30, so variations in the x-direction seem more pronounced than they actually are.

After the fluid fingers inside the high-permeability layers, it coarsens until a single broad finger remains in each layer (figure 12*b*). Figure 14(*a–d*) shows a sequence of snapshots of the concentration field with overlain streamlines for (*R*, *σ*, *Pe*, *n*) = (2, 0.1, 1000, 1) for which at intermediate times the flow is always in this channelling regime. In this example, *n* = 1 and so the channelling consists of a single pair counter-propagating fingers. The flow is predominantly longitudinal except in regions localized near large concentration gradients. Initially the streamwise velocity is large and localized in the finger. Upstream and downstream, where the viscosity is uniform, the velocity is imposed by the permeability, but is small relative to the contributions due to viscosity variations (figure 14*f* ). As time progresses, and the fluids become more mixed, the effect of the viscosity reduces and the streamwise velocity in the fingered region approaches the upstream and downstream velocity (figure 14*b–d*). The concentration deviations, figure 14(*e*), are uniform in the x-direction, vary sinusoidally in the y direction and are in phase with the velocity.

**FIGURE 15:**
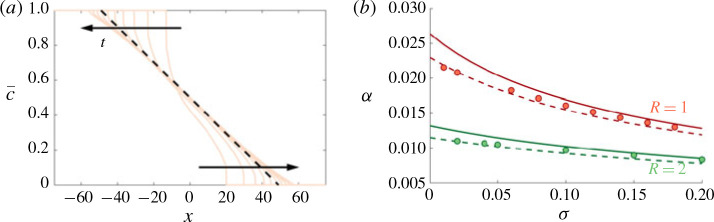
(Colour online) (*a*) Plot of $\bar{c}\left(x,t\right)$ from the numerical simulations (solid lines) and the theoretical prediction overlain (dashed line) for (*R*, *σ*, *Pe*, *n*) = (2, 0.1, 1000, 1). (*b*) Plot of the linear slope *α* of $\bar{c}$, as a function of *σ*, showing a least-squares fit to the data from numerical simulations (dots) and the theoretical solution (C 11) for *K* = 0.5 (solid line) and *K* = 0.6 (dashed line).

This behaviour resembles the late-time regime in the miscible viscous-fingering instability (i.e. when *σ* = 0). More specifically, the dynamics consists of an interior region where the background gradient is linear and steady and the ends of which are filled in by the propagating fingers (figure 15*a*). Superimposed on the steady background gradient are sinusoidal concentration deviations which decay in time.

In § C.2, we generalize the analysis from § 4.3.1 by allowing the deviations to evolve in time, and so derive a simplified model for the evolution of the mean concentration field in this regime. To test the validity of this model, we compare the predicted steady interior slope, *α*, to the results of the 2-D numerical simulations (figure 15*b*). As the viscosity ratio and permeability variance are increased α decreases due to the increased fluid velocity. The model shows reasonable agreement with the numerical simulations and the slight over-prediction of the slope is likely due to the underestimation of the velocity at early times.

### 6.3 Regimes III and IV: viscous fingering across layers

**FIGURE 16:**
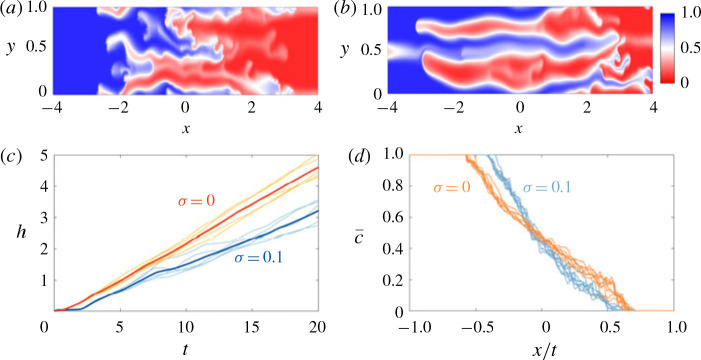
(Colour online) Evolution of the concentration field for |*R*| > σ and *R* > 0 in the viscous fingering across layers regime; (*R*, *Pe*, *n*) = (2, 2000, 32). (*a,b*) Colour maps of the concentration field for (*a*) σ = 0.1 and (*b*) σ = 0 at *t* = 8. (*c*) Plot of *h*(*t*) for five different simulations each (light, thin lines) and their average (dark, thick line). (*d*) Plot of $\bar{c}\left(x,t\right)$ versus the similarity variable *x/t* and *t* ranging from 6 to 20. Each curve corresponds to the average across five different simulations.

As noted earlier, if *n* > 1, the channelling regime can become unstable to a viscous-fingering instability which has fingers that are wider than the permeability structure imposed. The viscous fingers that develop in the heterogeneous medium are qualitatively similar to the fingers that develop in a homogeneous medium (figure 16*a,b*) and go through the same large-scale coarsening until a single finger remains. This single finger then evolves in manner similar to the single-finger state in a homogeneous medium (*σ* = 0).

One key difference between the two simulations is that in the heterogeneous medium there tends to be more tip splitting, aligned with the permeability field, due to velocity heterogeneities at the finger tips as well as fading and coalescence of fingers. The fact that the fingers are more unstable leads to more variability from simulation to simulation, more intermittent flow and non-uniform growth of the mixing length (figure 16*c*). The concentration field evolves in qualitatively the same manner in both cases, the profile is asymmetric and again has the similarity variable *x/t* (figure 16*d*). However the spreading rate is slower in the case when heterogeneities are present. Modelling the difference in spreading rate is the subject of future work.

Note that viscous fingering across layers is also possible when permeability effects dominate over viscous effects, *R* < *σ*. This can occur when the system reaches the late-time regime and the interface is still hydrodynamically unstable. For the interface to be stable, the width of the mixing zone must be *h* ∼ *RPe* (Nijjer *et al*. 2018). The mixing zone at the start of the late-time regime in the permeability-dominated scenario is *h* ∼ *Pe*Δ*u*/*n*^2^ ∼ *Pe*σ/*n*^2^ and so the late-time state may be unstable to further coarsening via a viscous instability if *R* > *σ*^2^/*n*^2^.

## 7 Discussion and conclusions

**FIGURE 17:**
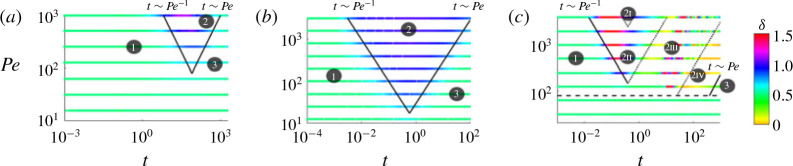
(Colour online) Representative plots of the scaling exponent of the mixing length, *δ*, found by locally fitting a power law of the form *h* = *At^δ^*, for three different parameter sets: (*a*) (*R*, *σ*, *n*) = (−2, 0.1, 1), demonstrating a viscously dominated stable displacement, as in § 5, (*b*) (*R*, *σ*, *n*) = (0.1, 1, 1), demonstrating a permeability-dominated displacement, as in § 4 and (*c*) (*R*, *σ*, *n*) = (2, 0.1, 6), demonstrating a viscously dominated unstable displacement, as in § 6. The regime boundaries (black lines) divide the early-time (regime 1), intermediate-time (regime 2) and late-time (regime 3) regimes. The dotted lines in (*c*) delineate the various intermediate-time regimes (§§ 6.1–6.3) and the dashed line delineates the hydrodynamically stable and unstable regions (Nijjer *et al*. 2018).

In this paper, we have examined miscible displacements in heterogeneous porous media. The main goal throughout this work has been to better understand the structure and evolution of the concentration field during stable and unstable displacement processes through the use of high-resolution numerical simulations. Motivated by the fact that many geological formations consist of layered sedimentary sequences, we considered porous media with a permeability structure that varies perpendicular to the flow direction. We considered cases where the injected fluid is equally viscous, more viscous or less viscous than the ambient fluid. In general we find that the flow evolves through three main flow regimes. Figure 17 summarizes these different possible regimes in the cases of viscously dominated stable displacements (figure 17*a*), permeability-dominated displacements (figure 17*b*), and viscously dominated unstable displacements (figure 17*c*). In each case the figure shows the instantaneous scaling exponent of the mixing length, *δ*, where *h* = At^*δ*^, for different *Pe*.

At early times (regime 1 in figure 17), the concentration field evolves through diffusion across an initially sharp interface (*δ* = 1/2) and is independent of both the log-viscosity ratio, *R*, and log-permeability ratio, *σ*(see § 3.1). Once advection begins to outpace diffusion (*t* ∼ *O*(1/*Pe*)) the flow transitions to the intermediate-time regime. At intermediate times (regime 2 in figure 17), the interplay between viscosity and permeability variations leads to a range of possible dynamics (*δ* ≠ 1/2). Finally, once the interface has become long and thin and transversely homogenized (*t* ∼ *O*(*Pe*)) the flow transitions to the late-time regime. At late times (regime 3 in figure 17), the flow becomes dominated by shear-enhanced dispersion and the concentration evolves diffusively again (*δ* = 1/2).

At intermediate times (regime 2 in figure 17), the interplay between viscosity and permeability variations leads to different behaviour depending on the relative size of *R* and *σ* and whether the injected fluid is more viscous or less viscous than the ambient fluid. When permeability effects dominate (*σ* > |*R*|; § 4); the viscosity modulates the effective permeability of the medium but otherwise evolves qualitatively in the same manner as when the two fluids have equal viscosity. When the injected fluid is more viscous than the ambient (|*R*| > *σ* and *R* < 0; § 5), the viscosity contrast tends to prevent channelling at the interface and reduces spreading of the two fluids relative to the equal viscosity, *R* = 0 case. When the injected fluid is less viscous than the ambient (|*R*| > *σ* and *R* > 0; § 6), a number of different possible regimes were identified (regimes 2I, 2II, 2III and 2IV in figure 17*c*). These different regimes arose due to the interplay between viscous fingering, which tends to cascade through a range of length scales, and the permeability structure, which has a fixed length scale. Overall, depending on which regime the flow is in, the permeability heterogeneity can either enhance or temper spreading relative to the uniform permeability case.

**FIGURE 18:**
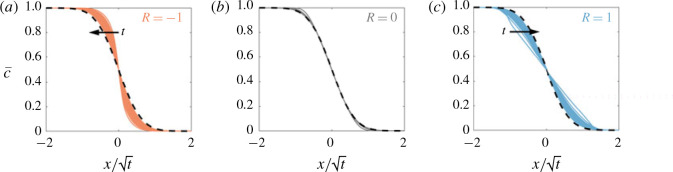
(Colour online) Long-time evolution of $\bar{c}\left(x,t\right)$ for *σ* = 0.3, (*Pe*, *n*) = (100, 1) and *t* ranging from 10 to 1000. The viscosity ratio is (*a*) stabilizing *R* = −1, (*b*) neutrally stable *R* = 0 and (*c*) de-stabilizing *R* = 1. The asymptotic, viscosity-independent solution, equation (3.4) with (3.11) is given by the dashed line.

At late times (regime 3 in figure 17), the concentration evolves through shear-enhanced dispersion (§ 4.3), which asymptotically becomes independent of the viscosity ratio and only depends on the permeability structure. Figure 18 demonstrates this behaviour: it shows the evolution of the concentration field for a neutrally stable displacement, large stabilizing displacement and a large de-stabilizing displacement. Although spreading is initially hindered (*R* < 0), or enhanced (*R* > 0), at late times they both tend to the same viscosity-independent self-similar solution. This means that for processes that occur over very small length scales or very long time scales, the viscosity difference becomes insignificant and the permeability structure dictates the rate of spreading and mixing of the two fluids.

In summary, the flow evolves through three distinct regimes: an early-time regime dominated by longitudinal diffusion, an intermediate-time regime dominated by longitudinal advection and a late-time regime dominated by shear-enhanced dispersion. Informed by high-resolution numerical simulations, we have developed simple models that capture the dominant physics in each of the regimes, which provide an easy way of quantitatively predicting the average behaviour of these systems.

## Figures and Tables

**FIGURE 19:**
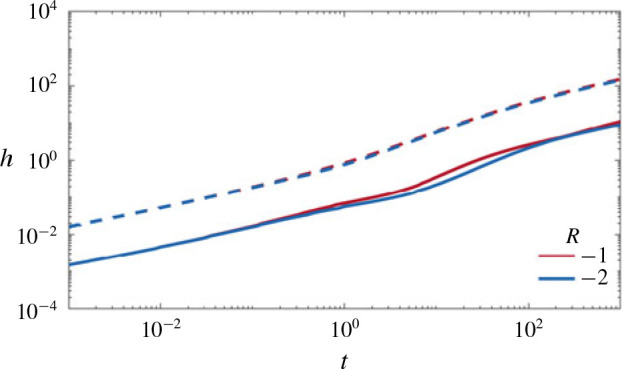
(Colour online) Plot of two different measures of *h* versus *t* for (*σ*, *Pe*, *n*) = (0.3, 300, 1) and *R* = −1, −2. The solid lines correspond to (2.18) and the dashed lines correspond to (A 1).

## Appendix A Comparison of mixing length metrics

Figure 19(*a*) shows a comparison of the mixing length quantity used throughout this paper (2.18) and the more typical measure

$$h=h^{*}={\left.x\right|}_{\bar{c}=0.01}-{\left.x\right|}_{\bar{c}=0.01}.$$ (A 1)

It can be seen that *h* calculated using (A 1) shows no discernible difference between the two simulations, whereas *h* calculated using (2.18) is noticeably different at intermediate times. This is because the simulations shown in figure 19 correspond to large stabilizing viscosities whose concentration profiles are composed of a sharp gradient at the interface, which depends on the viscosity ratio, and long tails upstream and downstream, which are independent of the viscosity ratio. Since (A 1) only picks up the growth of the tail regions, it is independent of *R*, whereas (2.18) accounts for the evolution of the sharp concentration gradient and hence depends on *R*. We therefore use (2.18) to measure the mixing length due to this sensitivity to the structure of the concentration field.

## Appendix B Asymptotic solutions for neutral displacements

When the variations in the permeability are small (*σ* ≫ 1), (3.2) becomes

Δu=2σ, (B 1)

such that at intermediate times the characteristic spreading velocity is

$$S={\int_{0}^{1}\left[\int_{0}^{y}\left(k\left(\eta \right)-1\right)d\eta \right]}^{2}\;dy≃\frac{\sigma^{2}}{8\pi^{2}}.$$ (B 2)

and at late times the shear-enhanced dispersivity, (3.12), is

$$\Delta u≃{\left(2\pi \sigma \right)}^{1/2}.$$ (B 3)

When the variations in the permeability are large (*σ* ≫ 1), the fluid velocity diverges at *y* = 1/2 and tends to −1 everywhere else. The charactersitic spreading velocity is

$$S={\int_{0}^{1}\left[\int_{0}^{y}\left(\left.\frac{e^{-\sigma \cos\left(2\pi \eta \right)}}{e^{\sigma }\left[{\left(2\pi \sigma \right)}^{-1/2}+0\left(\sigma^{-3/2}\right)\right]}\right|-1\right)d\eta \right]}^{2}\;dy.$$ (B 4)

At late times, the shear-enhanced dispersivity, *S* , is given by (3.12). Taylor expanding I_0_(*σ*) as *σ* → ∞, (3.12) becomes

$$S=\int_{0}^{1}{\left[-y+H\left(y-1/2\right)\right]}^{2}dy+0\left(\sigma^{-1}\right)=\frac{1}{12}+0\left(\sigma^{-1}\right).$$ (B 5)

Solving using Laplace’s method gives

$$S=\int_{0}^{1}{\left[-y+H\left(y-1/2\right)\right]}^{2}dy+0\left(\sigma^{-1}\right)=\frac{1}{12}+0\left(\sigma^{-1}\right).$$ (B 6)

## Appendix C Concentration models for *R* > σ

### C.1 Regime I: fingering within layers

To model the nonlinear evolution of the fingered region, we again assume the flow is in vertical flow equilibrium and that the streamwise velocity is given by (4.3). However, unlike in §§ 3.2 and 4.2 where the velocity depends on the permeability, here we assume the velocity only depends on the viscosity,

$$u=\frac{e^{Rc\left(x,y,t\right)}}{\int_{0}^{1}e^{Rc\left(x,s,t\right)}ds}-1,$$ (C 1)

since *σ* ≫ 1 Substituting the streamwise velocity into (2.16) and neglecting longitudinal diffusion gives

$$\frac{\partial \bar{c}}{\partial t}+\frac{\partial }{\partial x}\left(\frac{\int_{0}^{1}c\mu \left(c\right)dy}{\int_{0}^{1}\mu \left(c\right)dy}-\bar{c}\right)=0.$$ (C 2)

Next we consider *η_f_*(*x*) forward-propagating fingers of width *w_f_*(*x*) and *η_b_*(*x*) backward-propagating fingers of width *w_b_*(*x*). We make the simplifying assumption that the forward- and backward-propagating fingers have uniform concentration *c* = 1 and *c* = 0 respectively, but allow the viscosity to vary inside the finger. We have the additional constraints that the transversely averaged concentration is equal to the proportion of forward-propagating fingers $\eta_{f}w_{f}=\bar{c}$ and the total area of the fingers adds up to one, *η_f_w_f_* + *η_b_w_b_* = 1.

In a homogeneous medium (*σ* = 0), the forward- and backward-propagating fingers have viscosities $\mu \left(y\right)\approx e^{R\left(1-y^{2}/w_{f}^{2}\right)}$ and $\mu \left(y\right)\approx e^{R\left(y^{2}/w_{f}^{2}\right)}$ across the fingers respectively (see Nijjer *et al*. 2018). When *σ* is non-zero, the backward-propagating fingers are broad and coincide with the low permeability layers. In this case, the viscosity in the fingers is nearly uniform, *μ*(*y*) ≈ 1

Substituting our assumptions for the concentration and viscosity into (C 2) gives,

$$\frac{\partial \bar{c}}{\partial t}+\frac{\partial }{\partial x}\left(\frac{\eta_{f}\int_{-w_{f}}^{w_{f}}e^{R\left(1-y^{2}/w_{f}^{2}\right)}dy}{\eta_{f}\int_{-w_{f}}^{w_{f}}e^{R\left(1-y^{2}/w_{f}^{2}\right)}dy+\eta_{b}\int_{-w_{b}}^{w_{b}}1dy}-\bar{c}\right)=0.$$ (C 3)

This has the solution

$$\bar{c}\left(x,t\right)=\left\{\begin{array}{c} 1\\ \frac{1}{M_{e}-1}\left(\sqrt{\frac{M_{e}}{x/t+1}}-1\right)\\ 0 \end{array}\right.\begin{array}{c} x/t<\frac{1}{M_{e}}-1\\ \frac{1}{M_{e}}-1\leq x/t\leq M_{e}-1\\ x/t>M_{e}-1, \end{array}$$ (C 4)

Where

$$M_{e}=\frac{\sqrt{\pi }e^{R}erf\left(\sqrt{R}\right)}{2\sqrt{R}}.$$ (C 5)

The transversely averaged concentration evolves in exactly the same manner as in the homogeneous case but with a larger effective viscosity (cf. Nijjer **et al*.* 2018). This model prediction is given by the dot-dashed line in figure 13(*c*) and gives good quantitative agreement with the numerical simulations when *σ* ≳ 0.1.

### C.2 Regime II: stable channelling

This regime is characterized by a linear background gradient with superimposed deviations that decay. In § 5 we found that in the limit of large aspect ratio and small, quasi-statically evolving deviations, (2.17) reduced to (4.6). Here, in the channelling regime, the deviations decay exponentially, independently of the transversely averaged concentration. We therefore generalize the analysis of § 4.3 by allowing the deviations to evolve in time. Equation (2.16) remains the same, but (2.17) becomes

$$\frac{\partial c^{\prime }}{\partial t}+u\frac{\partial \bar{c}}{\partial x}=\frac{1}{Pe}\frac{\partial^{2}c^{\prime }}{\partial y^{2}}.$$ (C 6)

Again, the fluid flow is assumed to be in vertical flow equilibrium and so the velocity is given by (4.4). For simplicity, we will also assume *σ* ≪ 1, although the method below can be generalized to *σ* = *O*(1). Since $c^{\prime }\ll \bar{c}=O\left(1\right)$, (4.4) can be written, to leading order, as

$$u=Rc^{\prime }-\sigma \cos\left(2n\pi y\right),$$ (C 7)

and (C 6) becomes

$$\frac{\partial c^{\prime }}{\partial t}+R\bar{c}\frac{\partial \bar{c}}{\partial x}-\frac{1}{Pe}\frac{\partial^{2}c^{\prime }}{\partial y^{2}}=\sigma \cos\left(2n\pi \right)\frac{\partial \bar{c}}{\partial x}.$$ (C 8)

When *σ* = 0 this is exactly the late-time, single-finger exchange-flow behaviour of the miscible viscous-fingering instability (Nijjer *et al*. 2018). Solving (C 8) using separation of variables yields

$$c^{\prime }=\left(\frac{-\sigma \partial \bar{c}/\partial x}{\gamma }+Ke^{-\gamma t}\right)\cos\left(2n\pi y\right),$$ (C 9)

where $\gamma =R\partial \bar{c}/\partial x+4n^{2}\pi^{2}/Ρe$. The constant of integration *K* corresponds to the initial conditions at the onset of this regime and incorporates the nonlinear early-time spreading. A simple approximation would be that *K* ≈ 1/2 corresponding to the maximum amplitude of sinusoidally varying *c*′.

Depending on which term is dominant in (C 9), one gets one of two limiting solutions to (2.16). When *t* is small (intermediate times), the second term in (C 9) dominates over the first. The deviations are longitudinally uniform and decay exponentially, and the solution of (2.16) for $\bar{c}$ is

$$\bar{c}=\frac{1}{2}-\alpha x.$$ (C 10)

This tendency to a steady linear profile can be seen in figure 15(*a*). When *t* is large (late times), the first term dominates over the second. In this case the deviations decay quasi-steadily with the transversely averaged concentration. This case is exactly the small-*β* limit discussed in § 4.3.1 in the limit of small σ, and is dominated by shear-enhanced dispersion. In this case $\bar{c}$ tends to the self-similar error-function solution (3.4) with effective diffusivity (3.11). In addition, if *σ* > *R*, the first term, which scales like *O*(*σ*/*R*) (given $\partial \bar{c}/\partial x∼1/RPe$), is always larger than *Ke^−γt^*, consistent with the fact that this channelling behaviour is only present when viscosity variations are more important than permeability variations, *R* > *σ*.

Thus far the model is unable to predict the slope of the well-mixed interior region, *α*. To close the model, we relate the convective flux to the mixing that occurs. We consider a control volume containing everything to the right of the midplane. Neglecting longitudinal diffusion, the total convective flux into this control volume must balance the net change in concentration

$$\int_{0}^{T}\int_{0}^{1}uc^{\prime }dydt=\int_{0}^{\infty }\bar{c}dx\approx \frac{1}{8\alpha },$$ (C 11)

where *T* is a timescale marking the end of this regime. Assuming the flow is always in this channelling regime, we can substitute (C 7) and (C 8) into (C 11) to attain an implicit equation for *α*. This control volume approach inherently depends on the choice of *T*; as *T* → ∞, the transversely averaged concentration evolves via shear-enhanced dispersion and the estimate for *α* → 0. We therefore select *T* at the boundary between the intermediate- and late-time regimes such that the concentration profile is linearly well mixed in the interior and has not transitioned to the late-time diffusive solution. For our simulations, we find the choice *T* = 200 ≈ 3/*γ* appropriate, although we note that predictions are broadly insensitive to the choice of *T* as long as *T* ∼ *O*(1/*γ*).
